# Spontaneous Smoking Cessation in Parents

**DOI:** 10.1155/2021/5526715

**Published:** 2021-05-17

**Authors:** Emara Nabi-Burza, Richard Wasserman, Jeremy E. Drehmer, Bethany Hipple Walters, Mandy Luo, Deborah Ossip, Jonathan P. Winickoff

**Affiliations:** ^1^General Academic Pediatrics, Massachusetts General Hospital for Children, Boston, MA, USA; ^2^Tobacco Research and Treatment Center, Massachusetts General Hospital, Boston, MA, USA; ^3^Pediatrics, Larner College of Medicine, University of Vermont, Burlington, VT, USA; ^4^University of Rochester, Rochester, NY, USA; ^5^Richmond Center, American Academy of Pediatrics, Itasca, IL, USA

## Abstract

**Purpose:**

To determine the percentage of parents who report quitting spontaneously and examine the factors associated with these quits.

**Methods:**

As part of a cluster randomized control trial addressing parental smoking in a pediatric outpatient setting, 12-month follow-up survey data were collected from parents who had self-identified as smokers when exiting from 10 control practices. Parents were considered to have made a spontaneous quit if they reported not smoking a cigarette, even a puff, in the last 7 days and chose the statement “I did not plan the quit in advance; I just did it” when describing how their quit attempt started.

**Results:**

Of the 981 smoking parents enrolled at baseline, 710 (72%) completed the 12-month follow-up. Of these, 123 (17%) reported quitting, of whom 50 (41%) reported quitting spontaneously. In multivariable analysis, parents who reported smoking on some days vs. every day (OR 3.06 (95% CI 1.42, 6.62)) and that nobody had smoked in their home/car vs. someone had smoked in these settings in the past 3 months (OR 2.19 (95% CI 1.06, 4.54)) were more likely to quit spontaneously.

**Conclusions:**

This study shows that, of parents who quit smoking, a substantial percentage report quitting spontaneously and that intermittent smoking and smoke-free home/car policies are associated with reports of quitting spontaneously. Promoting smoke-free home/car policies, especially when parents are not willing to make a plan to quit smoking, might increase the likelihood that parents decide to quit without advance planning. Pediatric healthcare providers are uniquely positioned to use the child's visit to motivate parents to quit smoking and eliminate their child's exposure to tobacco smoke, regardless of the frequency of smoking or a readiness to plan a quit attempt. *Clinical Trial Registration*. This trial is registered with NCT01882348.

## 1. Introduction

Smoking tobacco remains one of the leading causes of mortality in the United States, accounting for more than 480,000 deaths annually [1–3]. The harmful effects of smoking on people exposed to tobacco smoke are also well documented [2, 4, 5]. Parental smoking is also associated with increased uptake of smoking by adolescents [6–10]. Earlier quitting dramatically lowers the risk of disease and increases the years of life gained from quitting [2, 11]. Smoking cessation is particularly important for parents, as parental smoking also exposes children to the harmful toxins via second- and thirdhand smoke [12–14].

To help parents quit smoking, child healthcare providers may be using behavioral stage-based interventions that largely follow the transtheoretical model of change [15, 16]. This model suggests that in quitting smoking, people typically go through various stages of motivation to quit; these stages include precontemplation, contemplation, preparation, action, and maintenance. Clinicians have used this model as a guide for conducting brief cessation interventions [17, 18]. However, studies have challenged this theory and concluded that stage-based interventions are no more effective than non-stage-based interventions in changing smoking behavior [19, 20].

Although planning is thought to be an important part of any behavior change, several studies have shown that a substantial proportion of smokers' quit attempts occur “spontaneously” or without planning [21–23]. These studies showed that such attempts can be as successful or more successful than planned quit attempts [21–25]. Some have questioned, however, whether these spontaneous quit attempts are truly unplanned or do include some level of planning or preparation [26, 27]. Further exploration of factors associated with spontaneous quit attempts is needed, especially among the understudied parent population, as their smoking impacts their children's health as well as their own. Child healthcare providers are uniquely positioned to identify parents who smoke and offer smoking cessation advice and treatment [28–30]. In doing so, they protect their children from the harms of tobacco smoke exposure [31].

To our knowledge, no studies have examined the rates of spontaneous quit attempts in the parent population. This paper is aimed at determining the percentage of smoking parents who report quitting spontaneously and examining the factors associated with these quits. Identifying these factors may help child healthcare providers tailor smoking cessation interventions to trigger “unplanned” quit attempts among parents.

## 2. Methods

The current analyses examined data collected from the control practices of the cluster randomized controlled trial, Clinical Effort Against Secondhand Smoke Exposure (CEASE). The trial tested the effectiveness of an intervention to systematically address parental smoking in the child healthcare office setting [32, 33]. Twenty child healthcare practices from 16 states were recruited for the trial from the Pediatric Research in Office Settings network, the practice-based research network of the American Academy of Pediatrics (AAP). The practices were randomized to control (usual care in which clinicians were not trained by the research team in smoking cessation intervention and practices were not supplied with any special materials to support cessation) and intervention study arms. The study protocol was approved by the Institutional Review Boards of Massachusetts General Hospital, Boston, MA, the AAP, and local practice institutional review boards, when required.

Research assistants at each child healthcare office collected baseline study data between June 2009 and March 2011. The research assistants administered a screening survey to all parents or legal guardians (henceforth mentioned as parents) who were exiting the office. Parents were eligible for enrollment if they indicated that they had smoked a cigarette, even a puff, in the last 7 days, were the parents or legal guardians of the child seen at the visit, were 18 years or older, were English speaking, and had a working telephone number. Eligible parents were offered the opportunity to consent and enroll in the study. The consent form specified that the study is aimed at understanding parental behaviors such as tobacco use and children's health. The enrollment surveys assessed smoking behaviors (past quit attempts, home and car smoking policies, and thirdhand smoke beliefs), smoking level (number of cigarettes smoked per day and frequency of smoking), and intention to quit (in the next 30 days and 6 months), as well as other demographic and visit-related questions (if they were asked about their smoking status, advised to quit, and discussed options to help quit smoking). Follow-up telephone surveys were conducted with all enrolled parents 12 months after baseline enrollment.

### 2.1. Statistical Analysis

The primary outcomes for the current paper were percentage of smoking parents who reported quitting spontaneously and factors associated with these spontaneous quits in the control arm. At the 12-month follow-up survey, parents who reported not smoking a cigarette, even a puff, in the last 7 days were considered to be quit [34, 35]. The following question [23, 36] was used to characterize each quit attempt as planned or unplanned: “Which of these 6 statements best describes how your most recent quit started?” Respondents were given the following options:

1. I did not plan the quit in advance; I just did it
2. I planned the quit for later the same day
3. I planned the quit the day beforehand
4. I planned the quit a few days to a few months beforehand
5. Other
6. Cannot remember

Parents were considered to have made a spontaneous quit if they chose the statement “I did not plan the quit in advance; I just did it.” Rates of parents who reported quitting spontaneously were compared with the rates of parents who were still smoking. Logistic regression was conducted to assess factors associated with spontaneous quits compared to those who did not quit. Variables that were significant (*p* < 0.10) in the bivariate analyses and those that had theoretical plausibility were added stepwise to a logistic regression model. A secondary analysis was conducted among those parents who reported quitting smoking to assess factors associated with spontaneous quits compared to planned quits. Variables that were significant (*p* < 0.10) in the bivariate analyses and those that had theoretical plausibility were added stepwise to a logistic regression model. The variance inflation factor (VIF) of the regression models was checked to ensure that the model did not have the problem of multicollinearity. All statistical analyses were conducted using Stata version 15 (Stata Statistical Software: Release 15, College Station, TX; StataCorp LLC).

## 3. Results

Of the 981 smoking parents enrolled at baseline in the control practices, 710 (72%) completed the telephone survey 12 months after enrollment. Of the 710 parents who completed the 12-month telephone survey, 123 (17%) reported not smoking a cigarette in the last 7 days. Of these, 41% (*n* = 50) reported their quit as being spontaneous. Most parents who quit spontaneously had children who were ≥10years (72%) and were covered by Medicaid (66%) (Table 1).

Bivariate analyses for parents who reported spontaneously quitting compared to those who did not quit showed that the following variables were associated with parents reporting a spontaneous quit attempt (*p* < 0.05): fewer cigarettes smoked per day (1-10 cigarettes/day vs. >10 cigarettes per day), lower frequency of smoking (some days vs. every day), and smoke-free home and car (no one smoking in the home and car of the parents vs. someone smoking in their home or car in the last 3 months). In the multivariable analysis (Table 2), parents who reported smoking on some days vs. every day (OR 3.06 (95% CI 1.42, 6.62)) and who reported that nobody had smoked in their home and car vs. someone had smoked in these settings in the past 3 months (OR 2.19 (95% CI 1.06, 4.54)) were more likely to have quit spontaneously compared to those who continued to smoke. The number of cigarettes per day was not significantly associated with parents quitting spontaneously. The mean VIF was 1.08, suggesting that multicollinearity was not a significant issue for the analysis.

Our results also showed that, of the 243 parents who smoked more than 10 cigarettes per day (heavy smokers) at baseline, 223 (92%) continued to smoke and only 20 (8%) reported quitting smoking at the 12-month follow-up. Among the heavy smokers who reported quitting at 12 months, 10 (50%) reported quitting spontaneously, 6 (30%) reported making planned quit attempts, and 4 (20%) did not remember how they quit.

In the multivariable logistic regression for the secondary analysis (Table 3), we found that parents who reported quitting spontaneously were less likely to have had the intention to quit in the next 6 months or 30 days compared to those who reported planning their quit (OR 0.18 (95% CI 0.03, 0.93)). The mean VIF was 1.09, suggesting that multicollinearity was not a significant issue for the analysis.

## 4. Discussion

The study results indicate that over 40% of all the parents who report quitting smoking one year after a visit to a child's healthcare practice report quitting spontaneously. Those parents who reported at the time of the visit that they smoked on some days vs. every day and that no one had smoked in their home and car in the past 3 months were more likely to have quit spontaneously. We also found that spontaneous quit rates were not lower among heavy smokers relative to light smokers who quit smoking.

Our findings are consistent with prior studies that have hypothesized that smokers who report making a spontaneous quit attempt maybe less dependent on smoking [21, 37]. Alternately, these smokers may be smoking on some days as a method of harm reduction and actually may be in the preparation stage of quitting [16, 38]. It is important to identify such parents and create awareness about the importance of smoking cessation and the use of evidence-based services in the form of nicotine replacement therapy and enrollment in the free state tobacco quitline to help them quit [32, 39].

Our findings show that people who had smoke-free homes or cars are more likely to report quitting spontaneously relative to those who do not quit. This finding is consistent with prior longitudinal and cross-sectional studies that have shown that smokers who created a smoke-free home are significantly more likely to make a quit attempt, quit, and smoke fewer cigarettes per day [40–44]. Smoke-free homes are also associated with reduced likelihood of adolescent smoking [45]. Studies have shown that interventions that target reduction in tobacco smoke exposure may be more successful in families who have young children and have emphasized the need of family-based interventions to help reduce the exposure of children to tobacco smoke [41]. Our findings raise the possibility that if child healthcare providers promote smoke-free homes and cars, it may enhance the likelihood of future spontaneous quitting.

Data from our study showed that the majority of people who smoke more than 10 cigarettes per day continue to smoke at 12 months, and of those who report quitting, half of them reported quitting spontaneously. It is noteworthy that the spontaneous quitting rate was not lower for heavy smokers who quit relative to light smokers.

We found that parents who reported they had quit spontaneously were less likely to have previously indicated having intentions to quit within a specific timeframe (6 months and 30 days) than those who had planned their quit in advance. However, our findings also demonstrate that having an intention to quit in the near future is not a prerequisite for successful smoking cessation. While many who successfully quit smoking do so by planning their quit date in advance, we found a substantial proportion of parents do so through spontaneous quits. More research is needed to better understand how spontaneous quits are triggered in smokers, yet clinician messaging might be more influential in encouraging quit attempts if it is tailored to match a parent's approach to quitting. Encouraging other beneficial tobacco control behaviors such as making homes and cars smoke-free might also increase the likelihood of prompting spontaneous quitting.

This study has several limitations. The secondary statistical analysis of data collected for the CEASE trial [32, 33] was not specifically powered for the research questions posed in this paper. Parental report of spontaneous quit attempts may have been overestimated or underestimated due to recall bias. The small sample size for the analysis to assess the factors associated with spontaneous quits vs. planned quits limited the likelihood of finding a difference in these two groups. Control group parents did not receive any intervention. Therefore, we cannot use this dataset to draw firm conclusions about tobacco cessation treatment or programs. Future studies should examine the ways in which tobacco cessation interventions might enhance spontaneous quitting.

Results are not representative of pediatric practices more broadly or the parental smoking population outside these practices. While we used a widely accepted seven-day point prevalence to assess current smoking status [35, 46] and the same measure to assess quitting at the 12-month follow-up, this item may not fully capture smoking status among those who smoke more infrequently than once a week. However, the majority of the smokers who quit spontaneously were at least daily smokers at baseline (62%). Despite these limitations, interpretation of the current results adds to the limited knowledge base about the prevalence of spontaneous quitting in the parental smoking population and factors associated with such spontaneous quits.

Most adult smokers want to quit smoking [47], and our research is consistent with previous research that shows that a significant proportion of quit attempts appear to be unplanned [22–24]. Child healthcare providers are uniquely positioned to use the teachable moment of a child's visit [48, 49] to motivate parents to quit smoking, as this may be a time when parents have increased likelihood of accepting smoking cessation assistance. They could use this opportunity to address parental smoking and help families become smoke-free using evidence-based cessation treatments. One evidence-based approach is offering specific pharmacological and behavioral cessation assistance [31, 50, 51].

## Figures and Tables

**Table 1:** Parents who reported quitting spontaneously vs. those who did not quit vs. those who reported planned quitting (total *N* = 680).

Characteristic	Overall sample (*N* = 680)	Spontaneous quits (*N* = 50, 7.4%), *N* (%)	Not quit (*N* = 587, 86.3%), *N* (%)	Planned quit (*N* = 43, 6.3%), *N* (%)	*p* value (spontaneous quit vs. not quit)	*p* value (spontaneous quit vs. planned quit)
Demographics						
Age					0.204	0.726
18-24 years	164 (24)	17 (34)	135 (23)	12 (28)		
25-44 years	459 (68)	30 (60)	402 (68)	27 (63)		
≥45 years	57 (8)	3 (6)	50 (9)	4 (9)		
Gender					0.70	0.940
Male	136 (20)	9 (18)	119 (20)	8 (19)		
Female	544 (80)	41 (82)	468 (80)	35 (81)		
Race and ethnicity					0.131	0.518
Hispanic	85 (13)	10 (20)	71 (12)	4 (9)		
Non-Hispanic White	443 (66)	30 (60)	381 (65)	32 (74)		
Non-Hispanic Black or African American	118 (17)	6 (12)	107 (18)	5 (12)		
Non-Hispanic others	29 (4)	4 (8)	23 (4)	2 (5)		
Education					0.886	0.405
<High school	95 (14)	6 (12)	85 (15)	4 (10)		
High school graduate	299 (44)	24 (48)	260 (44)	15 (36)		
Some college	181 (27)	14 (28)	154 (26)	13 (31)		
College graduate	103 (15)	6 (12)	87 (15)	10 (24)		
Youngest child's age					0.705	0.410
<1 year	1 (0)	0 (0)	0 (0)	1 (2)		
1-4 years	102 (15)	7 (14)	86 (15)	9 (21)		
5-9 years	70 (10)	7 (14)	60 (10)	3 (7)		
≥10 years	507 (75)	36 (72)	441 (75)	30 (70)		
Child's insurance coverage					0.911	0.879
Medicaid	427 (63)	33 (66)	368 (63)	26 (60)		
Private insurance/HMO	204 (30)	15 (30)	175 (30)	14 (33)		
Other/self-pay	44 (7)	2 (4)	39 (7)	3 (7)		
Smoking-related characteristics at baseline						
Frequency of smoking					0.000^∗^	0.756
Everyday	569 (84)	31 (62)	510 (87)	28 (65)		
Some days	111 (16)	19 (38)	77 (13)	15 (35)		
# cigarettes/day					0.011^∗^	0.441
1-10 cigarettes/day	440 (65)	40 (80)	364 (62)	36 (84)		
>10 cigarettes/day	240 (35)	10 (20)	223 (38)	7 (16)		
Quit readiness						
Consider quitting in 6 months	491 (75)	34 (72)	417 (73)	40 (93)	0.888	0.010^∗^
Seriously planning to quit in 30 days	292 (61)	21 (64)	241 (59)	30 (77)	0.619	0.014^∗^
Quit attempt in the past 3 months					0.390	0.041^∗^
Yes	335 (50)	27 (54)	276 (48)	32 (74)		
No	337 (50)	23 (46)	303 (52)	11 (26)		
Someone had smoked in their home and car in the past 3 months					0.000^∗^	0.565
Yes	502 (80)	24 (56)	455 (83)	23 (62)		
No	128 (20)	19 (44)	95 (17)	14 (38)		
Visit-related questions						
Asked about smoking	149 (22)	10 (20)	128 (22)	11 (26)	0.748	0.521
Advised to quit smoking	80 (12)	5 (10)	70 (12)	5 (12)	0.673	0.801
Discussed medicine to help quit smoking	16 (2)	3 (6)	12 (2)	1 (2)	0.079	0.384
Discussed programs to help quit smoking	4 (1)	0	4 (1)	0	0.554	n/a

**Table 2:** Multivariable logistic regression showing odds of parents reporting quitting spontaneously vs. those who did not quit (*N* = 578).

Variable	^a^OR (95% CI)
Frequency of smoking	
Some days	3.06 (1.42, 6.62)^∗^
Everyday	1.0^a^
Number of cigarettes smoked per day	
≥10 per day	0.50 (0.21, 1.19)
<10 per day	1.0^a^
Smoked in their home or car in the past 3 months	
No one smoked in their home and car	2.19 (1.06, 4.54)^∗^
Someone smoked in their home or car	1.0^a^
Age of the child	
≤4 years old	0.73 (0.27, 1.99)
5-14 years old	1.0^a^
Parent race	
White	1.79 (0.67, 4.82)
Non-White	1.0^a^
Parent gender	
Female	1.79 (0.67, 4.82)
Male	1.0^a^

^a^Reference group. ^∗^*p* < 0.05.

**Table 3:** Multivariable logistic regression showing odds of parents reporting quitting spontaneously vs. those who report planned quitting (*N* = 79)^∗^.

Variable	^a^OR (95% CI)
Intention to quit in the next 30 days or 6 months	
Yes	0.18 (0.03, 0.93)^∗^
No	1.0^a^
Age of the child	
≤4 years old	0.47 (0.12, 1.82)
5-14 years old	1.0^a^
Parent race	
White	1.69 (0.45, 6.35)
Non-White	1.0^a^
Parent gender	
Female	0.69 (0.18, 2.67)
Male	1.0^a^
Parent education	
<High school	2.12 (0.80, 5.65)
Some college/college graduate	1.0^a^

^a^Reference group. ^∗^Only parents who reported being quit at 12 months.

## Data Availability

Deidentified dataset will be available on request. Please email Jonathan P. Winickoff, MD, MPH (jwinickoff@mgh.harvard.edu), with your requests.

## Abbreviations

CEASE: Clinical Effort Against Secondhand Smoke Exposure
AAP: American Academy of Pediatrics
VIF: Variance inflation factor
OR: Odds ratio
CI: Confidence interval.
